# Hydrophobic tagging: A promising paradigm for targeted protein degradation

**DOI:** 10.1016/j.cellin.2025.100295

**Published:** 2025-12-04

**Authors:** Lilan Xin, Hongli Wang, Maoze Yang, Zhangxiao Guo, Man Zhu, Terry W. Moore, Chune Dong, Hai-Bing Zhou

**Affiliations:** a Department of Hematology, Zhongnan Hospital of Wuhan University, School of Pharmaceutical Sciences, Wuhan University, Wuhan 430071, Hubei, China; b Department of Pharmaceutical Sciences, University of Illinois Chicago, Chicago, IL 60612, United States; c Frontier Science Center for Immunology and Metabolism, State Key Laboratory of Virology and Biosafety, Provincial Key Laboratory of Developmentally Originated Disease, Key Laboratory of Combinatorial Biosynthesis and Drug Discovery (MOE) and Hubei Province Engineering and Technology Research Center for Fluorinated Pharmaceuticals, Wuhan University, Wuhan 430071, Hubei, China

**Keywords:** Targeted protein degradation, HyT, E3 ligase independency, Mechanism of action, Undruggable targets

## Abstract

Targeted protein degradation (TPD) has emerged as a groundbreaking therapeutic strategy, overcoming the limitations of traditional occupancy-driven pharmacology. Among TPD strategies, hydrophobic tag (HyT) technology exemplifies this paradigm shift by hijacking cellular protein quality control mechanisms for precise protein elimination. Structurally, HyT molecules integrate a target-specific ligand with a hydrophobic domain that emulates misfolded protein surfaces, facilitating selective recruitment of chaperone systems (e.g., heat shock protein 70, HSP70) and ubiquitin-proteasome system (UPS) activation, circumventing the E3 ligase dependency inherent to proteolysis-targeting chimera (PROTAC) systems. This innovative strategy offers distinct therapeutic benefits, including enhanced tissue-specific accumulation and the capacity to overcome resistance mechanisms. This review highlights the important advances in this rapidly growing field and critical limitations encountered in developing HyT degraders by analyzing the current status and representative examples of HyTs in degrading diverse pathogenic proteins, including oncogenic drivers (e.g., signal transducer and activator of transcription 3, STAT3), neurodegenerative aggregates (tau, α-synuclein), and viral envelope proteins. The critical developments, including the rational design of hydrophobic motifs and possible mechanistic insight into the degradation process of HyTs, have also been discussed.

## Graphical abstract

**Figure:**
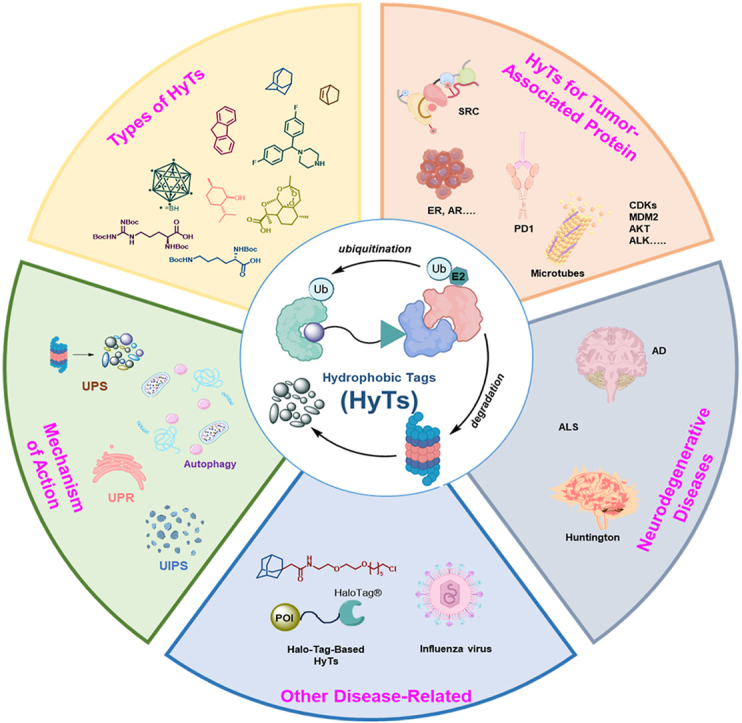

## 1 Introduction

During the evolution of oncological drug development, multiple targeted therapies have emerged, leveraging small-molecule inhibitors or monoclonal antibodies to inhibit tumor growth *via* mechanisms of receptor inhibition or modulating downstream signaling pathways. However, the prolonged use of these drugs could induce drug resistance in cancer cells, thereby diminishing their cytoprotective effects (Levenson et al., 1998; Salami & Crews, 2017). Currently, targeted therapies primarily rely on occupancy-driven mechanisms, which demand high dosages and frequent administration. This paradigm not only compromises patient compliance but also heightens selective pressure, precipitating the emergence of resistance. In contrast, targeted protein degradation (TPD) technology has emerged as a transformative paradigm, leveraging the ubiquitin-proteasome system (UPS) or autophagy-lysosome degradation pathways to achieve precise and selective degradation of specific proteins.

**Fig. 1:**
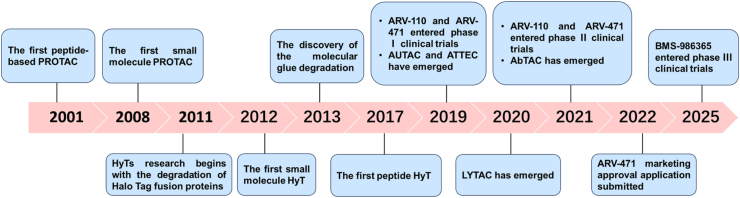
**Key discoveries and developments in the TPD**.

As an innovative strategy for addressing challenging drug targets, TPD technology enables specific recognition and direct degradation of pathogenic target proteins, harnessing the cell's intrinsic degradation machinery (Huang & Dixit, 2016). Among TPD technologies, proteolysis-targeting chimeras (PROTACs) (Neklesa et al., 2017), molecular glues (MGs) (Słabicki et al., 2020), hydrophobic tags (HyTs) (Gustafson et al., 2015), lysosome-targeted chimeras (LYTACs) (Banik et al., 2020), and autophagy-targeted chimeras (AUTACs) (Takahashi et al., 2019) have received significant attention. Since its inception in 2001, when Crews and Deshaies et al. initially reported the first peptide-based PROTAC (Sakamoto et al., 2001), this field has seen significant interest, as ARV-471 (Vepdegestrant), an estrogen receptor degrader, has been submitted to the FDA for new drug approval. Around the same time as the initial PROTAC reports, Crews et al. elucidated the pivotal role of HyTs in destabilizing the conformational stability of proteins, thereby facilitating their efficient lysosomal or proteasomal degradation (Fig. 1) (Lai & Crews, 2017; Neklesa et al., 2011; Tae et al., 2012).

**Fig. 2:**
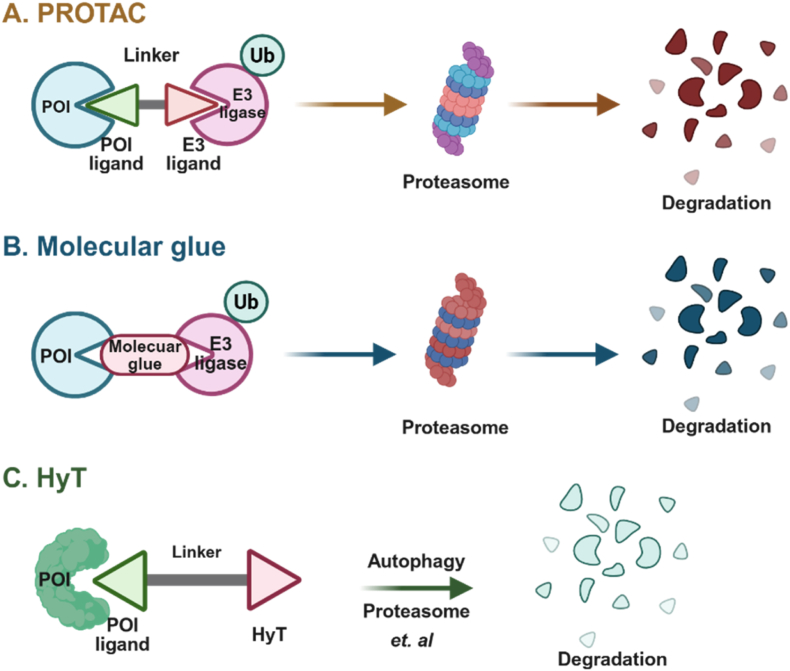
**Three main targeted protein degradation strategies.** (A) PROTACs recruit an E3 ligase to a POI through a bifunctional ligand with separate small-molecule recruiters joined together by a linker. (B) MGs operate through a similar ubiquitin-mediated process, but do not use a linker to join two discrete recruiters; rather, they bind to each unit, typically facilitating the formation of a ternary complex. (C) HyTs bind to a POI and are linked to a hydrophobic moiety that, through one of several mechanisms, causes degradation.

PROTACs consist of a target protein ligand, linker, and E3 ligand, which enables E3 ligases to bind to the protein of interest (POI), inducing ubiquitination and degradation (Fig. 2). MGs, small molecules that promote protein proximity, form ternary complexes to facilitate protein dimerization or co-localization. This modulates various biological and pharmacological effects by exploiting protein degradation pathways. Compared to PROTACs, MGs have smaller molecular weights and optimized physicochemical properties (Churcher, 2018; Gadd et al., 2017). They are particularly effective for targeting proteins inaccessible without direct binding pockets by disrupting or enhancing interactions between ubiquitin ligases and substrate proteins (Hughes et al., 2021). HyT is a bifunctional molecule comprising a lipophilic small molecule tag and a targeting ligand. Its molecular structure enables the destabilization of POI or the induction of a partially unfolded state, thereby triggering endogenous proteolysis (Fig. 2) (Wang et al., 2020).

HyT induces conformational changes in target proteins *via* hydrophobic interaction-driven mechanisms, thereby exposing degradation signals. In contrast to conventional PROTACs, which require stable ternary complex formation, HyT relies on hydrophobic effect-mediated physical interactions. This mechanism establishes a novel paradigm for targeted protein degradation, distinct from existing strategies. HyT exhibits substantial potential for degrading central nervous system proteins, offering therapeutic potential for neurological disorders (Tomoshige & Ishikawa, 2021). Through rational molecular design, HyT can be engineered to disrupt pathogenic protein-protein interaction (PPI) or target diseases driven by aberrant protein, circumventing key limitations of PROTACs and MGs. Mechanistically, HyTs do not depend on specific E3 ubiquitin ligases, significantly expanding their therapeutic scope. Compared to PROTACs, the advantages of HyTs are manifested in (1) a substantially lower molecular weight, enhancing cellular permeability and drug-like properties; (2) the elimination of teratogenic risks linked to Cereblon (CRBN)-thalidomide-based ligands; (3) greater structural versatility, enabling broader target coverage; and (4) a unique degradation mechanism, underscoring their great potential as a novel protein degradation approach (Wells & Kumru, 2024; Xie, Zhan, et al., 2023; Zhao et al., 2022).

To provide a clearer conceptual comparison among current degrader modalities, we outline the functional differences between HyTs, PROTACs, and MGs in Table 1.

**Table 1:** Functional comparison of HyTs, PROTACs, and MGs.

Feature	HyT	PROTAC	MG
**Typical molecular weights (MW)**	400–650 Da	700–1200 Da	300–500 Da
**CNS suitability**	High (due to low MW and permeability)	Low	Moderate–high
**Selectivity determinants**	Ligand affinity; conformational susceptibility	Ternary complex geometry	Dependent on PPI microenvironment (selectivity can be broad) (Chamberlain et al., 2014)
**Best suited target classes**	IDPs, multipass membrane proteins, organelle proteins (Neklesa et al., 2011)	Well-folded soluble proteins with ligandable pockets	Neo-substrates with glue-sensitive interfaces
**Advantages**	E3-agnostic initiation; access to Ub-independent degradation; CNS-permeable; eliminates proteins difficult for PROTACs	High selectivity; tunable ternary complex; mechanistically defined	Small, drug-like; can degrade traditionally undruggable targets
**Limitations**	Pathway heterogeneity; hydrophobicity-driven off-target effects at high dose	Limited by E3 ligase availability; poor PK; CNS impermeability	Hard to rationally design; dependence on subtle PPIs

## 2 Possible mechanistic insights into the degradation of HyT

**Fig. 3:**
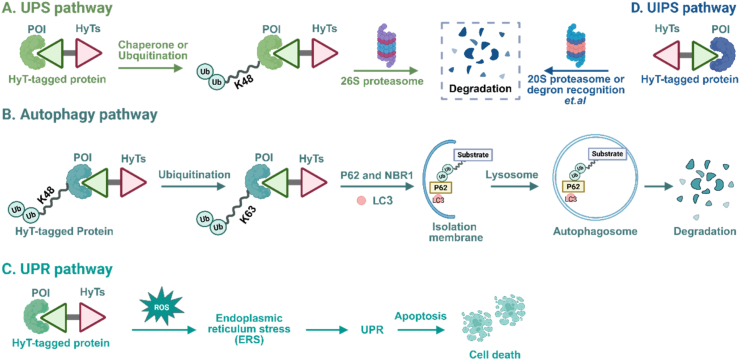
**Mechanisms of HyT-induced protein degradation** (He et al., 2023). (A) UPS pathway: E3 ligases recognize exposed hydrophobic motifs (or degrons) on surfaces of target proteins and catalyze the conjugation of polyubiquitin chains to these proteins through an E1-E2-E3 enzymatic cascade. The 26S proteasome degrades these polyubiquitinated targeted proteins, while DUBs remove polyubiquitin chains from substrates to recycle ubiquitin molecules, thereby sustaining subsequent ubiquitination cycles. (B) Autophagy pathway: DUBs remodel polyubiquitin chains on target proteins by hydrolyzing K48-linked chains and preserving K63-linked chains. Autophagy receptors p62/NBR1 recognize K63-polyubiquitinated proteins *via* ubiquitin-associated domains, recruit lipidated LC3-II on phagophores (isolation membranes) *via* LC3-interacting region (LIR) domains, and form a complex that promotes phagophore elongation and autophagosome biogenesis; subsequent fusion with lysosomes forms autolysosomes to degrade the POI. (C) UPR pathway: Endoplasmic reticulum activates the UPR; acute UPR promotes degradation of misfolded proteins (*via* ERAD/autography) and clearance of stressed endoplasmic reticulum components, whereas prolonged unresolved endoplasmic reticulum stress drives UPR to initiate apoptosis. (D) UIPS pathway: This pathway degrades oxidatively damaged/unfolded proteins *via* recognition of exposed hydrophobic regions, ATP-dependent unfolding, and 20S proteasomal cleavage, bypassing ubiquitination to maintain protein quality control.

The precise mechanism of action of HyT-based protein degradation (HyT-PD) remains elusive. HyT may facilitate POI degradation through multiple pathways. Normally, the hydrophobic domains of proteins are encapsulated within the protein structure; however, their exposure to the protein surface renders them unstable or misfolded proteins—a key signal recognized by the cellular chaperone system (e.g., Hsp70/Hsp90) and the UPS, or the autophagy-lysosome pathway (ALP), triggering the protein quality control (PQC) mechanism for degradation to prevent accumulation of toxic proteins (Jia & Han, 2023; Wunderley et al., 2014). Currently, four PQC mechanisms induced by HyT have been identified (Fig. 3), providing a novel insight into the mechanisms by which HyTs might function (He et al., 2023).

In eukaryotic cells, the UPS primarily promotes PQC by modifying exposed hydrophobic fragments with polyubiquitin chains through the action of the ubiquitin-activating enzyme (E1), ubiquitin-conjugating enzyme (E2), and ubiquitin ligase (E3) (Fredrickson et al., 2011). These modified proteins are then recognized and degraded by the 26S proteasome (Pohl & Dikic, 2019). Additionally, chaperone proteins like Hsp70 can present misfolded proteins to E3 for UPS-mediated degradation if folding fails (Chaudhuri & Paul, 2006).

Lysosomal autophagy constitutes a pivotal PQC pathway (Kwon & Ciechanover, 2017). When the UPS is overloaded, deubiquitinating enzymes (DUBs) remove K48 polyubiquitin linkages, allowing K63 polyubiquitin modification and lysosomal autophagy-mediated degradation (Kirkin et al., 2009; Mizushima, 2007; Yau & Rape, 2016). The endoplasmic reticulum, equipped with a rigorous quality control system, directly degrades misfolded proteins *via* the unfolded protein response (UPR) pathway (Schwarz & Blower, 2016). Accumulation of misfolded proteins in the endoplasmic reticulum activates the UPR and leads to protein elimination (Hetz, 2012). The ubiquitin-independent proteasome system (UIPS) is a rare PQC mechanism where the Boc_3_Arg tag targets POIs directly to the 20S proteasome for degradation, mimicking partially unfolded proteins without the need for the 19S proteasome or ubiquitin (Long et al., 2012). Glickman et al. found that C-degrons promote ubiquitin-independent proteasomal degradation, which exerts regulatory/quality control functions, with ubiquilin family proteins mediating ubiquitin-independent proteasomal degradation substrate turnover (Makaros et al., 2023). Greenberg, Elledge, et al. found that Midnolin binds the proteasome *via* its C-terminal α-helix, activates proteasomal activity through the N-terminal ubiquitin-like domain (UBL), and recognizes exposed β-strands of nuclear protein substrates *via* the central Catch domain to form intermolecular β-sheets, directly delivering substrates to the proteasome for degradation—bypassing ubiquitination to enable selective degradation of these nuclear proteins (Gu et al., 2023).

Compared to UPS-mediated PROTAC degradation, multi-mechanism HyT-PD introduces new avenues for protein design targeting multiple degradation pathways. Employing the fulvestrant (Ful)-induced surface hydrophobicity strategy (see below for details), HyT-PD achieves efficient target protein degradation by attaching hydrophobic molecules (such as adamantane or Boc_3_Arg) to their surfaces, mimicking the partially unfolded protein state (Neklesa et al., 2017).

Generally, HyT-induced degradation can be conceptually divided into an E3-agnostic initiation step and pathway-specific downstream processing. In the initiation phase, the hydrophobic moiety destabilizes the POI and exposes hydrophobic surfaces, which are recognized as misfolded independently of any specific E3 ligase. Subsequently, depending on the cellular context, chaperone systems and PQC factors route the substrate toward distinct degradation fates. UPS-mediated degradation is characterized by K48-linked polyubiquitination, dependence on E1/E2/E3 activity, and sensitivity to 26S proteasome inhibitors. In contrast, UIPS-mediated degradation persists under E1/E2/E3 inhibition and is typical for intrinsically disordered proteins (IDPs) (Ukmar-Godec et al., 2020). When HyT-modified proteins accumulate as larger aggregates (Mizushima & Komatsu, 2011) or membrane-associated complexes (Dikic & Elazar, 2018), the autophagy pathway often predominates, involving K63-linked ubiquitin chains, p62/NBR1 recruitment, LC3-positive autophagosomes, and sensitivity to lysosomal inhibitors. Additionally, for ER-localized or secretory-pathway proteins, HyT-induced misfolding may activate the UPR, which subsequently channels substrates toward ER-associated degradation (ERAD, UPS-dependent) or autophagy, serving as an upstream stress-sensing mechanism within the PQC network (Chen et al., 2023).

## 3 HyT degradation

In 2011, Craig M. Crews unveiled HyT technology, a pioneering subset within the realm of protein degradation methodologies (Neklesa et al., 2011). The core of HyT lies in its ability to mimic misfolded proteins, attracting chaperone proteins or proteasomes to degrade the POI. Compared to PROTAC technology, HyT boasts a smaller molecular weight, superior druggability, robust degradation capacity, broader applicability, and avoidance of teratogenicity risks associated with thalidomide derivatives. Various HyTs, including adamantane (Gustafson et al., 2015), fluorene (Go et al., 2020), pyrene (Hachisu et al., 2016), carborane (Asawa et al., 2021), and norbornene (Xie, Zhan, et al., 2023), have been successfully developed, achieving effective POI degradation for targets, such as human epidermal growth factor receptor 3 (HER3), estrogen receptor (ER), androgen receptor (AR), enhancer of zeste homolog 2 (EZH2), and poly ADP ribose polymerase (PARP).

PROTAC technology harnesses the dumbbell structure of heterobifunctional small molecules, with one terminus binding to the POI and the other recruiting the E3 ubiquitin ligase to form a ternary complex. This ubiquitination process facilitates proteasomal-mediated degradation, offering a novel therapeutic approach for “undruggable” proteins inaccessible to traditional small-molecule inhibitors. Analogous to PROTAC molecules, the HyT degrader consists of three components: the HyTs, the linkers, and the POI ligands (He et al., 2022). For PROTACs, the E3 ligase ligands, the linkers, and the POI ligands are all crucial for degradation efficacy (Paiva & Crews, 2019). As a bifunctional TPD molecule, HyT integrates lipophilic tags with target protein ligands for precise targeting.

### 3.1 The types of HyT moieties

**Fig. 4:**
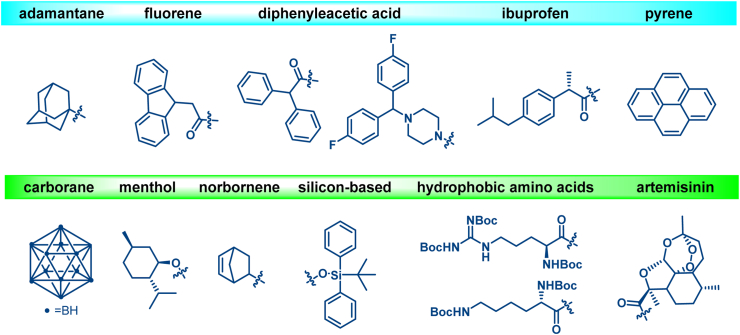
**Types of existing HyT moieties**.

The repertoire of currently developed HyTs primarily comprises adamantane (Gustafson et al., 2015), fluorene (Go et al., 2020), carborane (Asawa et al., 2021), menthol (Li et al., 2022), norbornene (Xie, Zhan, et al., 2023), hydrophobic amino acids (Ma, Wang, et al., 2024; Wang et al., 2025), artemisinin (Qing et al., 2025), and so on (Fig. 4). These HyTs exhibit distinct biochemical and biophysical properties. Adamantane moieties are favored due to their exceptional stability and cellular membrane permeability. Fluorene-based HyTs demonstrate potent degradative capabilities, particularly when conjugated with PARP inhibitors. The carborane structural motif, characterized by alternating CH and B units, displays extraordinary stability, icosahedral geometry, and pronounced hydrophobicity and serves as an effective pharmacophore, interacting with target proteins to facilitate proteasome-mediated degradation. Meanwhile, menthol, norbornene, and hydrophobic amino acid-based HyTs, each possessing unique attributes such as high oral bioavailability and degradation efficiency, mediate distinct proteasomal degradation pathways. Nevertheless, given a diverse range of POIs, the exploration of additional HyT types may be essential to optimize degradation efficacy.

### 3.2 The types of linkers

**Fig. 5:**
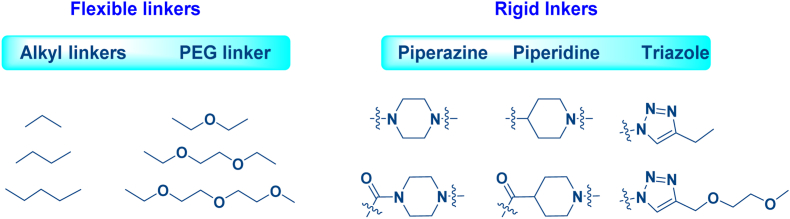
**Types of linkers in HyTs**.

The linker of HyT-PD, analogous to its role in PROTACs, is crucial for protein degradation efficiency, physicochemical properties, and pharmacokinetic parameters. Optimal degradation requires a linker length that strikes a balance between excessive or insufficient lengths. Furthermore, different HyT types demand unique optimal linker lengths for conjugation. The linker structures, comprising flexible alkyl linkers (carbon chains and PEG chains) and rigid linkers (piperazine, piperidine, and triazole), exert a profound impact on HyT-PD's absorption, metabolic stability, and molecular conformation (Fig. 5) (Bemis et al., 2021; Cyrus et al., 2011; He et al., 2023). The affinity of HyT-PD for a POI is a crucial determinant of degradation efficiency, while the correlation between binding affinity and degradation potency is complexly interwoven with additional factors, including cellular membrane permeability and compound stability (Hachisu et al., 2016). In light of these findings, optimizing HyT-PD necessitates a holistic consideration of linker length and structure to augment degradation capability.

## 4 Applications of HyT in drugs discovery

### 4.1 Targeted protein degradation by HyTs for tumor-associated protein inhibition

#### 4.1.1 ER

**Fig. 6:**
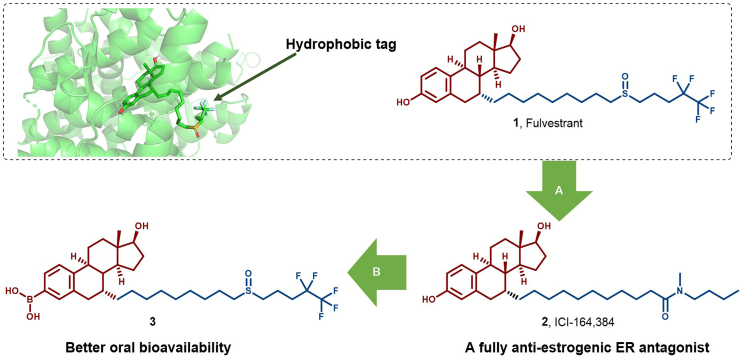
**Molecular structures of steroidal SERD (1–2) and their analog (3).** (A) Early discovery of an ER degrader based on Ful, showing that Ful binds to the ligand-binding pocket of ERα (PDB: 2BJ4). (B) To improve the oral bioavailability of Ful, SERD analog **3** (an ER degrader) was discovered by replacing the phenol with a boronic acid (Liu et al., 2016).

The relationship between hydrophobic structures and protein degradation can be traced to the steroidal selective estrogen receptor down-regulators (SERDs) Ful (**1**) and ICI-164,384 (**2**), which are estradiol scaffolds modified for high affinity (Fig. 6). By introducing long alkyl chains, these compounds, upon binding to the ER, induce helix 12 (H12) to undergo disordering, exposing the hydrophobic patch on the ER that is susceptible to degradation *via* the ubiquitin-proteasome pathway (Wakeling et al., 1991). Notably, **2**, a fully anti-estrogenic ER antagonist, lacks an agonistic effect in ER^+^ tissues (Wakeling, 1989; Wakeling and Bowler, 1987, 1988a, 1988b). Its long 7α-side chain interacts with the coactivator binding groove, thus exhibiting full anti-estrogenic effects. Conversely, a side chain with 13–14 carbon atoms may exhibit ER agonist activity (Bowler et al., 1989; Hilmi et al., 2012; Traboulsi et al., 2017). The side chain of **2** effectively disrupts the ER's H12-ligand binding domain (LBD) linkage, preventing agonistic or antagonistic conformations, thereby highlighting its complete anti-estrogenic effect (Pike et al., 2001b).

Structural analysis of the ER complex with **2** suggests that exposure of a large hydrophobic group on **2** to the protein surface may trigger UPR, thereby leading to ubiquitination and proteasomal degradation (Pike et al., 2001a; Wijayaratne & McDonnell, 2001). The hydrophobic alkyl structure in Ful subtly alters the ER structure, causing it to be recognized as misfolded and subsequently degraded *via* the proteasomal pathway. To improve the oral bioavailability of Ful, modification led to the generation of SERD analog **3** by introducing a boronic acid moiety (Fig. 6) (Burslem & Crews, 2017). Specifically, Wang's group discovered the analog **3**, which induced the degradation of ERα in T47D cells (Liu et al., 2016).

**Fig. 7:**
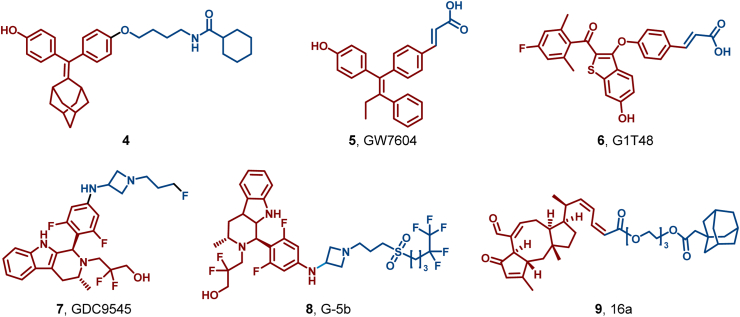
**Molecular structures of ER HyTs (4, 9) and non-steroidal SERDs (5–8)**.

In 2018, based on the ER ligand discovered in his lab, Sharma's group conjugated a series of hydrophobic moieties and found **4** as an efficient SERD against ERα (DC_50_ = 0.4 nM) (Wang et al., 2018). Non-steroidal SERDs can be categorized into two primary subclasses: acrylic side chain SERDs and basic side chain SERDs. Acrylic side chain SERDs, including GW7604 (**5**) and G1T48 (**6**), constitute a potent class of anti-estrogenic agents that exhibit no cross-resistance to 4-hydroxytamoxifen (Fig. 7). Crystallographic analysis of ERα in complex with GW7604 (**5**) revealed that interaction between the carboxylic acid of **5** and the ER peptide backbone led to the conformational change that exposes the receptor's hydrophobic surface to UPS-mediated degradation. Basic side chain SERDs, such as GDC-9545 (**7**), have advanced to clinical trials (Hurvitz et al., 2023; Lawson et al., 2023). These compounds exhibit excellent oral bioavailability and significant antitumor activity in preclinical models of acquired endocrine tolerance, particularly in estrogen receptor 1 (*ESR1*) mutation and cyclin-dependent kinase 4/6 (CDK4/6) inhibitor-resistance models. However, their efficacy did not surpass that of Ful, leading to their clinical discontinuation.

In 2023, Wang et al. designed G-5b (**8**) by incorporating a fluorine-substituted hydrophobic fragment of Ful into the scaffold of the new-generation oral SERD GDC-9545, which is currently undergoing Phase III clinical trials (Fig. 7). **8** exhibited tenfold antiproliferative efficacy compared to Ful (IC_50_ = 9.6 ± 0.7 nM, DC_50_ = 1.0 ± 0.2 nM) and efficiently degraded ER *via* the proteasomal pathway (IC_50_ = 1.0 ± 0.1 nM, DC_50_ = 0.4 ± 0.1 nM), while also demonstrating the ability to inhibit ER mutants resistant to Ful. **8** thus presents a promising solution to address the challenges associated with Ful treatment (Wang et al., 2024).

Furthermore, based on the marine sesquiterpene compound MHO7 (16a, **9**), Liang et al. designed novel SERDs by introducing a long hydrophobic chain into MHO7 derivatives (Fig. 7). Notably, **9** emerged as a promising candidate, exhibiting an IC_50_ value of 0.41 μM against the ER^+^ breast cancer cell line MCF-7 (Liang et al., 2022).

#### 4.1.2 AR

**Fig. 8:**
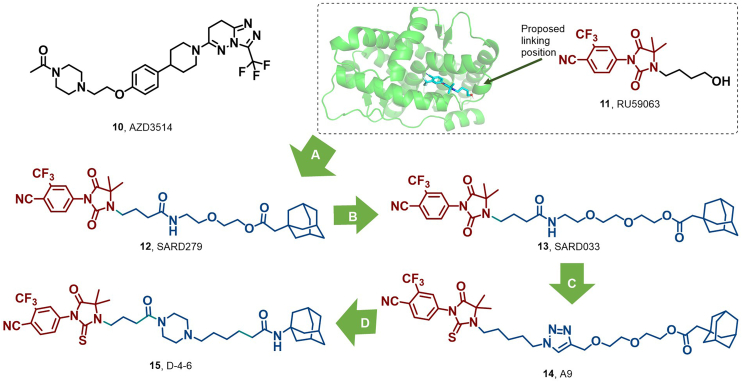
**Molecular structures of SARD (10), AR antagonist (11), and AR HyTs (12–15).** (A) Early discovery efforts of AR degrader based on RU59003 and adamantyl, and the crystal structure of **11** in the binding pocket of AR (PDB: 2PIP) (Osguthorpe & Hagler, 2011). (B–D) Non-oral AR degraders **12**–**15** were discovered by replacing different linkers (Sun, Wang, Li, Li, et al., 2024; Teutsch et al., 1994; Xie et al., 2020).

AR serves as a pivotal nuclear transcription factor in the pathogenesis of prostate cancer (PC). Endogenous androgens, particularly dihydrotestosterone, exert their biological effects through binding to AR. Androgen deprivation therapy (ADT), which involves reducing androgen levels, represents the standard treatment option (Gao et al., 2005; Ha et al., 2022). While ADT is efficacious in its early stages, most patients develop resistance within two years, culminating in castration-resistant prostate cancer (CRPC) (Bradbury et al., 2011). The first selective androgen receptor degrader (SARD), AZD3514 (**10**), is an oral AR inhibitor that decreases cellular AR levels (Fig. 8) (Bradbury et al., 2013). The underlying mechanism driving AR protein downregulation remains incompletely characterized, involving multiple pathways distinct from the hydrophobic tagging or protein destabilization observed with SERDs (Loddick et al., 2013). Despite entering phase 1 clinical trials, **10** did not advance due to poor pharmacokinetics (PK) and adverse effects (Omlin et al., 2015). Meanwhile, the lack of a precisely defined degradation mechanism could introduce potential safety risks for current HyT drugs.

In 2015, Crews et al. successfully synthesized SARD279 (**12**) and SARD033 (**13**) by conjugating the AR antagonist RU59063 (**11**) to an adamantyl moiety *via* a PEG linker (Fig. 8) (Gustafson et al., 2015; Teutsch et al., 1994). Notably, **12** selectively induced AR degradation in lymph node carcinoma of the prostate (LNCaP) cells, suppressed AR target gene expression, and inhibited cell proliferation. Particularly, in the presence of competitive AR antagonists, **12** showed superior efficacy compared to enzalutamide. Furthermore, both **12** and **13** exhibited inhibitory effects on the proliferation of enzalutamide-resistant prostate cancer cell lines. This study provided the first validation of an adamantane-based strategy *via* non-covalent HyT-mediated interactions, paving the way for broadening the scope of this approach.

In 2020, Ke et al. conjugated a HyT to an AR ligand, yielding compound A9 (**14**), which induces the UPS-mediated AR degradation (Fig. 8) (Xie et al., 2020). Compound **14** exhibited potency in AR degradation in LNCaP cells with an IC_50_ of 1.75 μM, arrested the LNCaP cell cycle at the G0/G1 phase, and induced mild apoptosis.

In 2024, Shan et al. reported HyT D-4-6 (**15**), demonstrating significant AR protein degradation activity, achieving degradation rates of 57% and nearly 90% following 24 h of treatment at 5 μM and 20 μM, respectively (Fig. 8). Compound **15** exhibited a notable inhibitory effect on LNCaP cell proliferation with an IC_50_ of 4.77 μM, establishing a foundation for the development of novel therapeutic agents targeting metastatic castration-resistant prostate cancer (mCRPC) (Sun et al., 2024).

#### 4.1.3 Steroid receptor coactivator-1 (SRC-1)

**Fig. 9:**
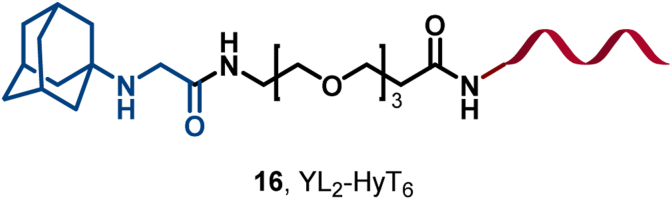
**Molecular structure of SRC-1 HyT (16)**.

The aberrant activation of SRC-1, a pivotal coactivator within the transcriptional network, is correlated with cancer progression (Lee et al., 2017; Nikolovska-Coleska et al., 2004; Oñate et al., 1995; Xu et al., 2009). Leveraging the crystal structure of YL2 bound to the PAS-B domain of SRC-1, Choi et al. developed YL2-HyT6 (**16**), a potent degrader of SRC-1, which markedly decreased SRC-1 levels and invasiveness in MDA-MB-231 cells (Fig. 9) (Choi et al., 2021).

#### 4.1.4 Murine double minute 2 (MDM2)

**Fig. 10:**
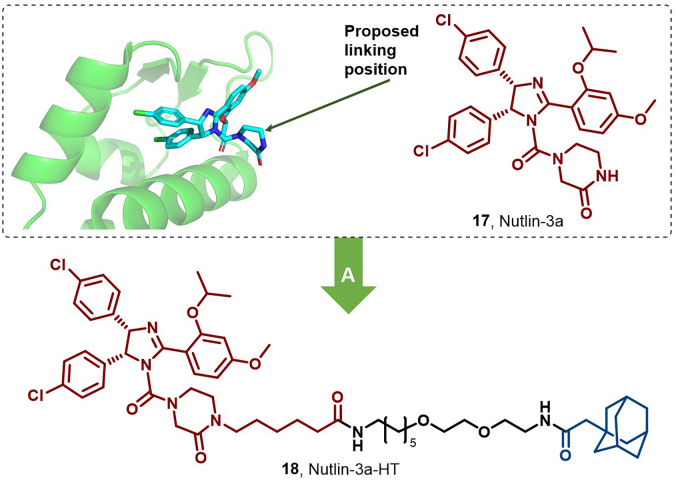
**Molecular structure of MDM2 HyT (18).** (A) MDM2 degrader **18** was achieved by linking the MDM2 inhibitor (**17**) with an adamantyl group, and the crystal structure of **17** in the binding pocket of MDM2 (PDB: 4J3E).

Loss of function of the tumor suppressor P53 is a crucial factor in cancer progression. MDM2 overexpression facilitates P53 degradation by binding to its transactivation domain. Nietzold et al. developed Nutlin-3a-HT (**18**), an MDM2-targeting compound with a HyT, which efficiently degraded MDM2 and induced a higher level of apoptosis in HCT-116 cells compared to nutlin-3a (**17**) (Nietzold et al., 2019). These findings underscore the broad potential of HyT technology in protein degradation and offer novel strategies for cancer therapy (Fig. 10).

#### 4.1.5 Cell cycle-dependent kinases (CDKs)

As pivotal members of the serine/threonine kinase family, CDKs play a fundamental role in cell cycle regulation by phosphorylating essential substrates, thereby facilitating DNA synthesis and mitosis (Wu et al., 2020). However, deregulated CDK activation frequently results in cell cycle disruption and hyperproliferation, fostering oncogenesis (Martin et al., 2017). Consequently, targeting CDKs has emerged as a crucial strategy in anticancer drug development.

**Fig. 11:**
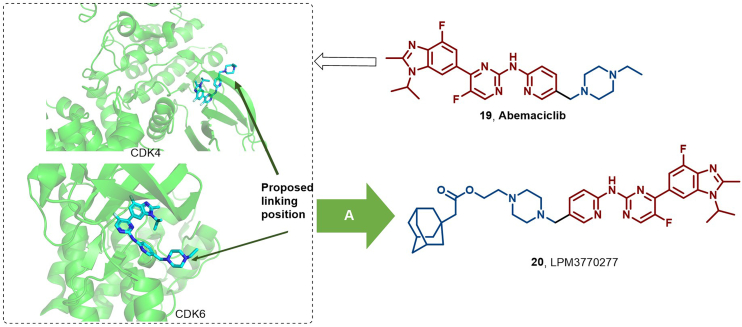
**Molecular structures of CDK inhibitor (19) and CDK HyT (20).** (A) CDK degrader **20** was achieved by appending abemaciclib (16) with an adamantyl group; the crystal structures of 16 in the binding pocket of CDK4 (PDB: 7SJ3) (Gharbi et al., 2022) and CDK6 (PDB: 5L2S).

To address the challenges of resistance and off-target toxicity associated with small molecule kinase inhibitors that directly impede catalytic function, researchers propose modulating the non-catalytic functions of kinases as a novel therapeutic direction. Notably, technologies like PROTACs, HyTs, and MGs offer promising avenues to explore these non-catalytic functions (Martin et al., 2017). Illustratively, in 2022, Tian et al. identified LPM3770277 (**20**), a HyT degrader derived from abemaciclib (**19**) by incorporating an adamantyl moiety, which effectively binds to and degrades CDK4/6 proteins *via* proteasomal degradation and lysosome-mediated autophagy (Fig. 11). This compound demonstrated superior efficacy and safety against triple-negative breast cancer (TNBC) compared to the clinically approved abemaciclib (Qiu et al., 2022). The bioactivity of CDKs is closely associated with cyclins, forming functional heterodimeric complexes that regulate key cellular processes, including cell proliferation, differentiation, apoptosis, and DNA damage repair (Tadesse et al., 2015). While CDK levels remain constant throughout the cell cycle, cyclin levels are dynamically regulated by protein synthesis and proteasomal degradation, ensuring temporal CDK activation (Cao et al., 2014).

The specific targeting of CDK2, CDK4, CDK6, CDK9, and CDK12 using PROTACs has shown promising therapeutic potential by targeting CDK complexes and their associated cell cycle regulatory proteins concurrently, although the precise mechanistic underpinnings remain incompletely elucidated (Jiang et al., 2019; Olson et al., 2018). Concurrently, MGs have been applied to enhance CDK12-DDB1 interaction, resulting in reduced cyclin levels. However, the development of a systematic, structure-based design strategy for these glues remains an unmet challenge (Lv et al., 2020; Mayor-Ruiz et al., 2020; Słabicki et al., 2020).

**Fig. 12:**
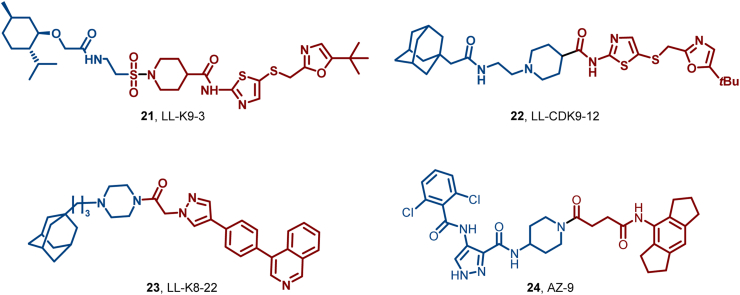
**Molecular structures of CDKs-HyTs 21–24**.

Zhou et al. designed LL-K9-3 (**21**), a hybrid molecule that selectively and simultaneously degrades CDK9 and cyclin T1. This compound exhibited significant inhibitory effects on AR- and Myc-driven oncogenic transcriptional programs compared to monovalent CDK9 PROTAC (Fig. 12) (Li et al., 2022). Lin et al. introduced LL-CDK9-12 (**22**), a novel dual degrader that significantly enhanced antiproliferative activity and disrupted downstream signal transduction in prostate cancer cells (Fig. 12) (Lin et al., 2023). Zhou et al. further utilized HyT technology to develop LL-K8-22 (**23**), achieving the selective and simultaneous degradation of CDK8 and its interacting cyclins. Notably, this HyT-based degrader demonstrated a more profound suppression of CDK8-mediated downstream signaling pathways (Fig. 12) (Wang et al., 2023). More recently, Liu et al. reported the first ATG101-recruiting selective CDK9 degrader, AZ-9 (**24**), which achieved potent, selective *in vitro*/*in vivo* CDK9 degradation (with Cyclin T1), affected downstream phenotypes, and acted by recruiting ATG101/LC3 to trigger autophagy-lysosome degradation (Zhong et al., 2025).

Collectively, these studies not only underscore the potential of HyT technology in targeting traditionally undruggable proteins but also exhibit distinct advantages over PROTACs in the simultaneous degradation of CDK-cyclin complexes.

#### 4.1.6 Anaplastic lymphoma kinase (ALK)

**Fig. 13:**
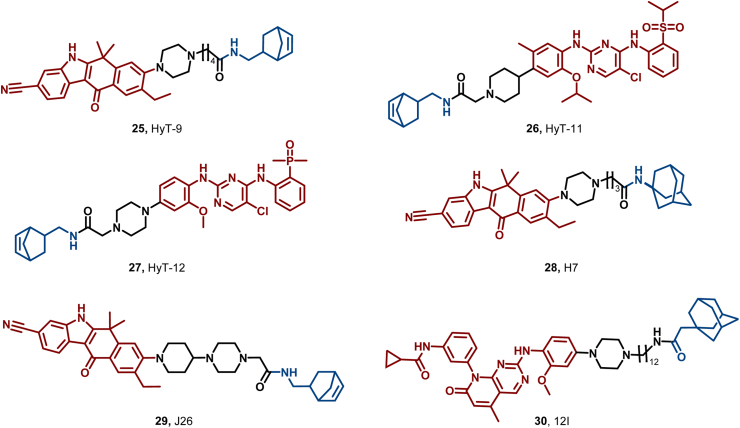
**Molecular structures of ALK HyTs (25–29), and Akt HyT (30)**.

ALK, a pivotal member of the insulin receptor kinase subfamily, has emerged as a prime target for various malignancies (Lemmon & Schlessinger, 2010). Currently, five ALK inhibitors (crizotinib, ceritinib, alectinib, brigatinib, and lorlatinib) have demonstrated clinical efficacy (Xie, Zhu, et al., 2023). Nonetheless, research into PROTAC targeting ALK remains active. PROTAC Q2, reported by Xu et al., encountered issues of limited cell permeability and oral bioavailability due to its high molecular weight (Xie et al., 2021). To address these limitations, they utilized HyT technology to introduce the norbornene structure, a low-molecular weight HyT, thereby enhancing the pharmacokinetic properties of the PROTAC and enabling the development of a novel ALK-targeting degrader (Xie, Zhan, et al., 2023). HyT-9 (**25**) showed significant antiproliferative activity and degradation capacity both *in vitro* and *in vivo*, with moderate oral bioavailability. Furthermore, this team designed HyT-11 (**26**) and HyT-12 (**27**) by incorporating the norbornene moiety into two other ALK inhibitors, thus establishing a novel strategy for ALK degradation (Fig. 13). Then, Zhu et al. further developed an adamantane-based HyT degrader (H7, **28**) targeting ALK, which exhibited enhanced antitumor activity (Zhu et al., 2025). Additionally, Xu et al. reported a novel ALK-targeting HyT degrader (**29**) that overcomes E3 ligase-dependent drug resistance, enabling efficient degradation of ALK in drug-resistant tumor models (Xie et al., 2025).

#### 4.1.7 Protein kinase B (AKT)

AKT, an enzyme belonging to the serine/threonine kinase family and also known as protein kinase B, plays a pivotal role in cellular processes, such as cell growth, proliferation, survival, metabolism, and migration (Gao et al., 2014). However, the structural similarity among the three AKT homologs (AKT1, AKT2, and AKT3) complicates the development of selective inhibitors (Gonzalez & McGraw, 2009).

Ding et al. performed computational analysis of the solvent-exposed surfaces of XTF-262, an AKT conjugate modified with adamantyl groups, with a focus on the distinct lysine distributions in the conformational regions of AKT isoforms (Xu et al., 2022). They successfully reported the first isoform-selective AKT3 degrader, 12l (**30**) (Fig. 13). In osimertinib-resistant H1975 (H1975OR) NSCLC cells, **30** demonstrated robust proteasomal degradation of AKT3 and anti-tumor effects without affecting AKT1/2. This discovery not only reveals a noncanonical role of AKT3 in NSCLC cell survival but also offers a novel therapeutic strategy to overcome drug resistance and achieve selective AKT3 degradation, highlighting the immense potential of HyT technology in developing protein-selective degraders.

#### 4.1.8 Polo-like kinase 1 (Plk1)

**Fig. 14:**
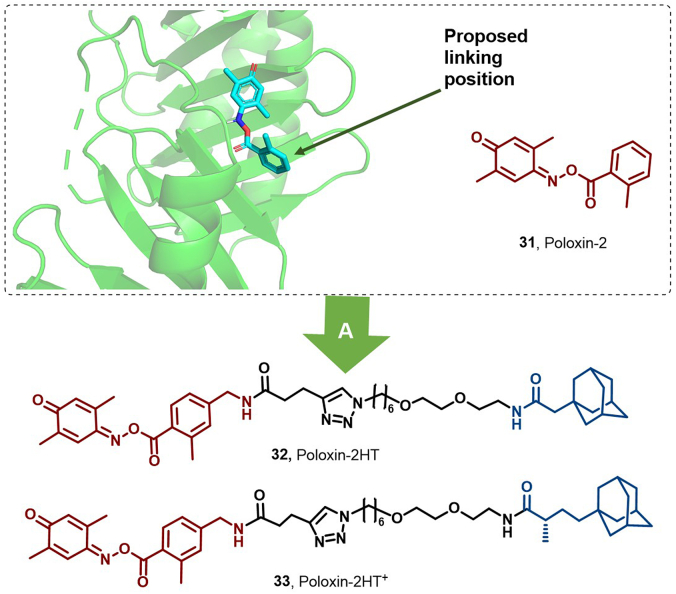
**Molecular structures of Plk1 HyTs (31–33).** (A) P1k1 degraders **32** and **33** were obtained by linking Plk1 inhibitor Poloxin-2 (**31**) with adamantyl *via* distinct linker chains, and the crystal structure of **31** in the binding pocket of Plk1 (PDB: 4HY2).

PIK1, a crucial regulator of cellular mitosis, is a potential target for cancer therapy. However, its inhibitors face challenges in competing with blood ATP for binding (Elia et al., 2003; Strebhardt, 2010). Scharow et al. identified poloxin as the first small-molecule Plk1 inhibitor (Poloxin-2, **31**) and further enhanced its antiproliferative effects *via* the development of Poloxin-2HT (**32**), partly due to Plk1 degradation (Scharow et al., 2015). Subsequently, Poloxin-2HT^+^ (**33**) demonstrated stronger inhibition of cell activity and induction of apoptosis, hinting at a potential role of the linker moiety in promoting PIK1 degradation (Fig. 14) (Rubner et al., 2019).

#### 4.1.9 Microtubules

Microtubules, a vital component of the eukaryotic cytoskeleton, are integral to cell division, signal transduction, shape maintenance, and vesicular transport. They consist of α- and β-subunit dimers, and imbalances in their dynamics disrupt cell division mechinery, rendering microtubules a key target in anticancer therapy (Gasic & Mitchison, 2019).

**Fig. 15:**
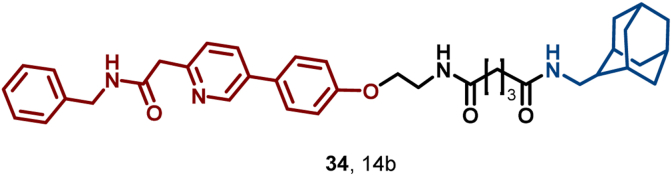
**Molecular structures of microtubule HyT (34)**.

In 2024, Wang et al. reported a novel microtubule protein-targeting drug, compound 14b (**34**), which exerts dual mechanisms of action: inhibiting microtube polymerization and inducing microtubule protein degradation (Fig. 15). **34** not only suppressed the polymerization of purified recombinant microtubule proteins but also degraded α- and β-microtubules in MCF-7 cells. Additionally, **34** exhibitd potent antitumor activity in a 4T1 xenograft model, achieving a tumor growth inhibition (TGI) rate of 74.27% in this model (Sun et al., 2024).

#### 4.1.10 Janus kinase (JAKs)

The JAK family comprises non-receptor tyrosine protein kinases JAK1, JAK2, JAK3, and TYK2. Cytokine binding to its receptor activates JAKs, initiating regulatory signals. JAK3 is specific to myeloid and lymphoid systems, endothelial and vascular smooth muscle cells, whereas other JAKs are ubiquitous (Hu et al., 2021).

**Fig. 16:**
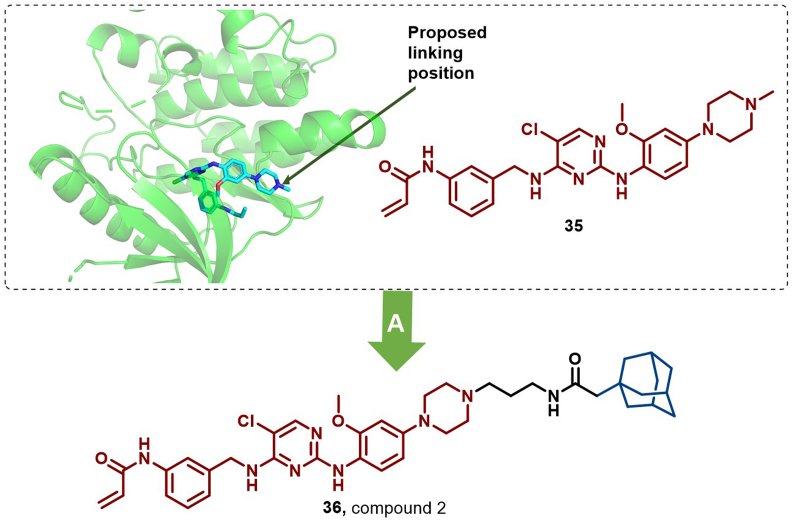
**Molecular structure of JAK3 HyT (36).** (A) JAK degrader **36** was achieved by linking to **35** with the adamantyl moiety, and the crystal structure of **35** in the binding pocket of JAK (PDB: 4V0G).

In 2015, Gray et al. developed compound 2 (**36**), a HyT-based JAK3 degrader, by conjugating **35** to an adamantyl moiety (Fig. 16). Compound **36** exhibited potent and selective JAK3 inhibition *via* distinct mechanisms involving JAK3 degradation and additional inhibitory pathways (Tan et al., 2015).

#### 4.1.11 Phosphodiesterase δ (PDEδ)

Kirsten rat sarcoma viral oncogene homolog (KRAS), encoded by the KARS gene, is closely linked to cancer development and progression, thus emerging as a highly promising target for antitumor drug development (Cox et al., 2014; Kessler et al., 2019; Waters & Der, 2018). PDEδ modulates KRAS function by regulating its cellular membrane localization (Chen et al., 2019). Disrupting the KRAS-PDEδ interaction disrupts KRAS trafficking and distribution, interfering with the KRAS signaling pathway and effectively inhibiting tumor cell proliferation (Klein et al., 2019; Liu et al., 2019).

**Fig. 17:**
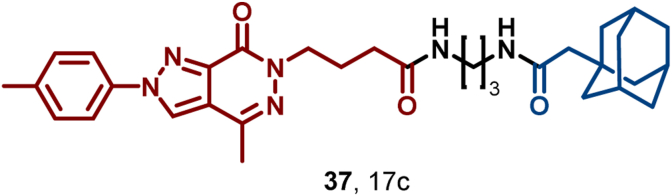
**Molecular structures of PDEδ HyT (37)**.

Guo et al. successfully designed and synthesized the first HyT-based degrader 17c (**37**) targeting PDEδ, which not only demonstrated a high PDEδ degradation rate but also remarkable antitumor ability (Fig. 17) (Guo et al., 2022). In SW480 cells, **37** achieved dose-dependent PDEδ degradation with a DC_50_ of 11.4 μM and a sustained activity lasting up to 24 h. Compared to the traditional PDEδ inhibitor deltazinone, **37** demonstrated superior antitumor capacity.

#### 4.1.12 Proprotein convertase subtilisin-like/kexin type 9 (PCSK9)

**Fig. 18:**
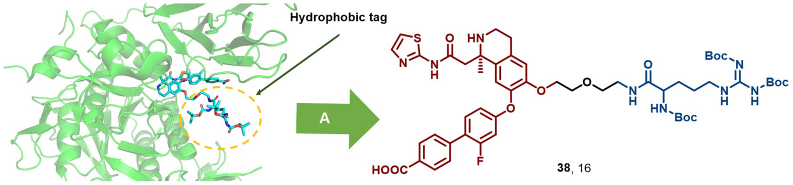
**Molecular structure of PCSK9 HyT (38).** A: The crystal structure of compound **35** with PCSK9 protein (PDB: 6U26).

PCSK9 is a key protein in atherosclerosis progression. The development of conventional small-molecule drugs has faced significant obstacles due to the long-standing dominance of *anti*-PCSK9 monoclonal antibodies (Sabatine, 2019). In 2020, Petrilli et al. combined Boc_3_Arg with a screened PCSK9 high-affinity ligand, yielding compound 16 (**38**) that effectively degraded PCSK9 (DC_50_ = 3.4 μM). This HyT-based degrader exhibited potential utility for atherosclerosis (Fig. 18) (Petrilli et al., 2020).

#### 4.1.13 Epidermal growth factor receptor (EGFR)

EGFR, a receptor tyrosine kinase located on the cell membrane, plays a pivotal role in cell growth and differentiation. EGFR expression is abnormally elevated and closely associated with malignant behaviors such as tumor growth, invasion, and metastasis in NSCLC, thereby becoming a crucial target for targeted therapy (To et al., 2019). Currently, various EGFR-targeted drugs, including gefitinib, icotinib, afatinib, and osimertinib, have been developed.

**Fig. 19:**
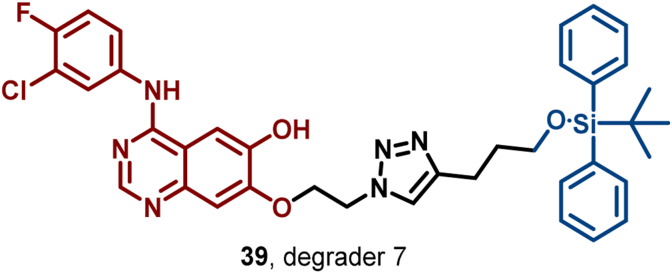
**Molecular structure of EGFR HyT (39)**.

Recently, Yang et al. reported a novel EGFR degrader, degrader 7 (**39**), which is based on the silicon-based HyT (SiHyT) strategy and constructed by conjugating gefitinib to a TBDPS silyl ether moiety (Fig. 19) (Ma, Zhang, et al., 2024). This degrader exhibited potent EGFR degradation activity, along with superior metabolic stability and oral bioavailability compared to traditional carbon-based HyTs. Furthermore, the SiHyT strategy has been successfully extended to the targeted degradation of programmed death-ligand 1 (PD-L1) and BTK, further exploiting the vast potential of HyTs in TPD technology.

#### 4.1.14 Programmed death protein 1 (PD-1)

PD-1 and its ligand PD-L1 constitute vital immune checkpoints implicated in tumor-induced immunosuppression, where PD-L1 expression facilitates tumor evasion from the host immune system. Blocking the PD-1 and PD-L1 interaction enables tumor-reactive T cells to overcome regulatory barriers and elicit potent antitumor responses (Dermani et al., 2019).

**Fig. 20:**
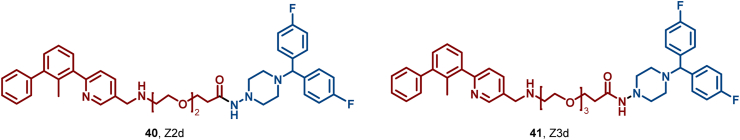
**Molecular structures of PDL1 HyTs (40–41)**.

In 2024, Gao et al. developed a novel HyT-based degrader incorporating a 4,4′-bifluorobenzhydrylpiperazinyl moiety for PD-L1 degradation. Compounds Z2d (**40**) and Z3d (**41**) effectively degraded PD-L1 *via* the proteasomal pathway across various cell lines, including NCI-H460 and H-1080, highlighting the potential to develop HyT-based degraders for other therapeutic targets (Fig. 20) (Gao et al., 2024).

#### 4.1.15 HER3

**Fig. 21:**
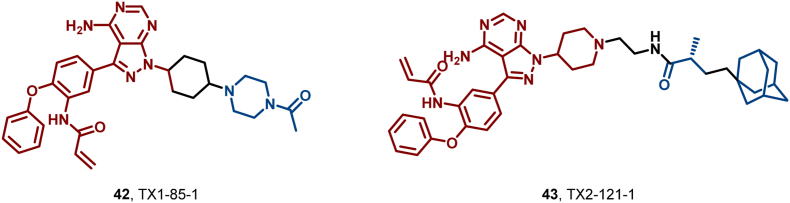
**Molecular structures of HER3 ligand (42) and HER3 HyT (43)**.

HER3, an undruggable pseudokinase in the human EGFR family, holds a pivotal role in numerous aggressive human malignancies, especially breast, ovarian, and non-small-cell lung cancers (NSCLC) (Baselga & Swain, 2009; Lee-Hoeflich et al., 2008; Tanner et al., 2006; Vaught et al., 2012). Given its absence of kinase activity and ligand binding sites, no small-molecule inhibitors targeting HER3 have been reported to date. In 2015, Gray et al. (Xie et al., 2014) achieved a breakthrough by introducing the first selective and irreversible HER3 ligand, TX1-85-1 (**42**), which covalently binds to Cys721 of HER3, exhibiting potent binding with an IC_50_ value of 23 nM. Subsequently, they developed TX2-121-1 (**43**) by appending an adamantane fragment to the piperidine moiety of **42**, converting it into a HER3-degrading HyT derivative (Fig. 21). HyT **43** retains a high affinity for HER3 (IC_50_ = 49.2 nM) and efficiently degrades HER3 *via* Hsp70/Hsp90-mediated chaperone pathways and the proteasomal degradation pathway, thereby inhibiting downstream signaling and exhibiting antiproliferative effects with an EC_50_ in the range of 0.8–1.4 μM. Despite the limitations of covalent interactions for clinical application, HyT technology offers a new direction for targeting and degrading traditionally undruggable targets such as HER3 and EZH2.

#### 4.1.16 EZH2

**Fig. 22:**
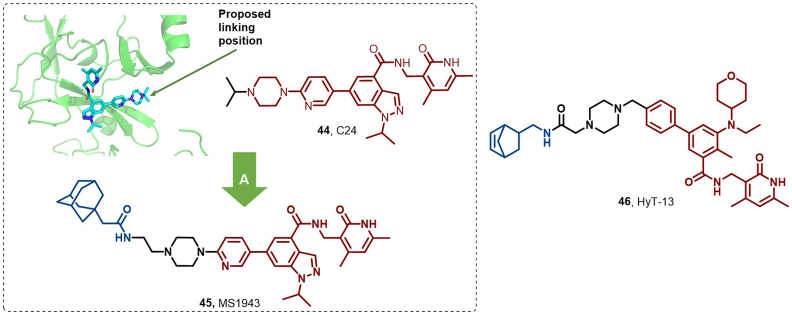
**Molecular structures of EZH2 HyTs (45–46).** (A) EZH2 degrader **45** was achieved by EZH2 inhibitor **44** with adamantyl, and the crystal structure of **44** in the binding pocket of EZH2 (PDB: 4MI0).

In another study, EZH2, a key histone methyltransferase in triple-negative breast cancer (TNBC), regulates transcriptional silencing and other gene expression programs by catalyzing trimethylation of histone H3 lysine 27 (Cao et al., 2002; Kuzmichev et al., 2002). Given the therapeutic limitations of EZH2 inhibitors in TNBC and the beneficial downregulation of EZH2 by siRNA (Curry et al., 2015; Kuzmichev et al., 2002), reducing EZH2 protein levels is crucial for TNBC treatment. In 2019, Ma et al. successfully synthesized the HyT-based degrader MS1943 (**45**) starting from the EZH2-selective inhibitor C24 (**44**), demonstrating its ability to enhance EZH2 degradation *via* the proteasomal pathway (Fig. 22) (Ma et al., 2020). Notably, **45** demonstrated robust antitumor activity and high oral bioavailability *in vivo*, suggesting a new direction for TNBC clinical treatment.

Concurrently, Xu et al. designed the EZH2 degrader HyT-13 (**46**) by conjugating the EZH2 inhibitor tazemtostat with norbornene (Fig. 22) (Xie, Zhan, et al., 2023). This HyT-based degrader utilizes a unique mechanism of action, inducing destabilization of POI and recruiting Hsp70, rather than directly binding to the proteasome for target protein degradation.

#### 4.1.17 Histone deacetylases (HDACs)

HDACs, epigenetic enzymes that remove acetyl groups from histones and other proteins, have been implicated in numerous malignancies due to their dysregulation. Although extensive research has focused on PROTACs targeting HDACs, there is a relative lack of studies on inhibition of HDAC by HyT technology (Sangwan et al., 2018).

**Fig. 23:**
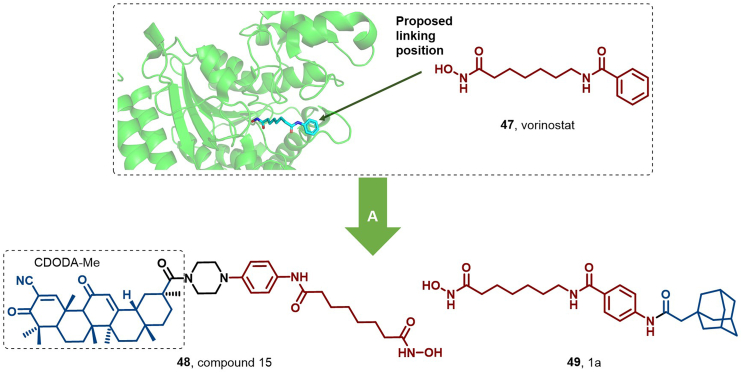
**Molecular structures of HDAC3/6 HyT (48) and HDAC1 HyT (49).** A: HDAC degraders (**48** and **49**) were achieved by linking the HDAC inhibitor (**47**) with CDODA-Me or adamantyl, and the crystal structure of **47** in the binding pocket of HDAC1 (PDB: 4LXZ).

In 2019, Zhao et al. synthesized compound 15 (**45**) by integrating the pharmacophore of vorinostat (**44**) with the semisynthetic 18β-glycyrrhetinic acid (GA) analog, methyl-2-cyano-3,11-dioxo-18β-olean-1,12-dien-30-oate (CDODA-Me) (Fig. 23). Although **45** displayed weaker HDAC inhibitory activity compared to vorinostat, it was more potent than CDODA-Me at reducing HDAC3 and HDAC6 protein expression levels. Notably, **45** exhibited favorable pharmacokinetic properties, including a prolonged half-life of 16.75 h (Huang et al., 2020).

In 2023, Feller et al. designed two series of compounds using vorinostat as a ligand, incorporating diverse linkers and HyT groups. These compounds effectively degrade HDAC *via* the UPS-mediated pathway. Notably, compounds 1a, 2a, 2d, and 3a demonstrated superior antiproliferative activity against the MM.1S cell line compared to vorinostat. Western blot analysis revealed that compound 1a (**46**) exhibited the highest apoptosis-inducing capability and effectively degraded HDAC1 (Fig. 23) (Feller & Hansen, 2023).

#### 4.1.18 Protein arginine methyltransferases (PRMT)

The protein arginine methyltransferase 6 (PRMT6) family members are key enzymes in arginine methylation, with PRMT6, a type I PRMT, playing a key role in processes such as gene expression, DNA repair, and cellular signaling. PRMT6 regulates gene expression and exerts diverse effects on various cancers by modulating cell growth, migration, invasion, apoptosis, and drug resistance, positioning it as a promising antitumor therapeutic target (Chen et al., 2022).

**Fig. 24:**
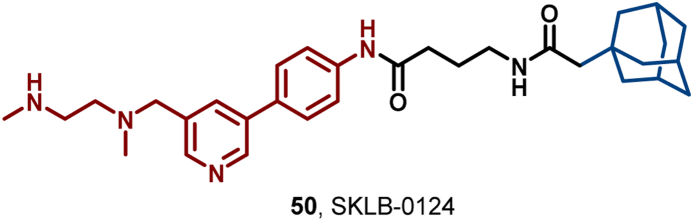
**Molecular structure of PRMT6 HyT (50)**.

In 2024, Yang et al. introduced SKLB-0124 (**50**), a first-in-class PRMT6 degrader utilizing HyT methodology (Fig. 24). Importantly, **50** selectively degraded PRMT6 through the UPS and potently inhibited the proliferation of HCC827 and MDA-MB-435 cells, with IC_50_ values of 5.9 μM and 5.1 μM, respectively. Furthermore, it effectively induced apoptosis and cell cycle arrest in these cell lines (Yang et al., 2024).

#### 4.1.19 Glioma-associated homolog-1 (Gli1)

Gli1, a transcriptional effector of the Hedgehog signaling pathway, is aberrantly activated in various cancers, promoting proliferation, survival, angiogenesis, metastasis, metabolic rewiring, and chemoresistance (Avery et al., 2021).

**Fig. 25:**
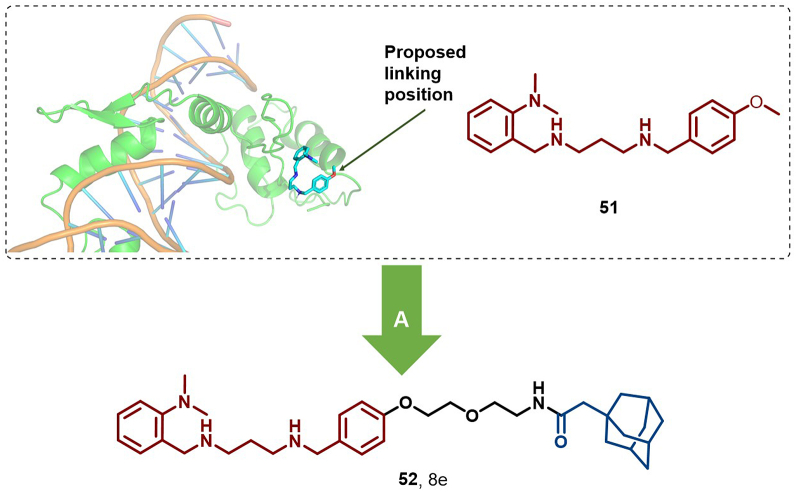
**Molecular structure of Gli1 HyT (52).** A: Gli1 degrader **52** was achieved by linking the Gli1 inhibitor with an adamantyl moiety, and the crystal structure of **51** in the binding pocket of Gli1 (PDB: 2GLI).

In 2023, Li et al. introduced the first HyT-based Gli1 degrader, **8e** (**52**), which effectively inhibited proliferation and induced apoptosis in Gli1-overexpressing HT29 cells (IC_50_ = 0.46 μM, DC_50_ = 5.4 μM), outperforming GANT 61D (**51**) in antiproliferative activity (Fig. 25). Moreover, **52** disrupted canonical and noncanonical Hedgehog signaling and overcame resistance to Smoothened antagonists (Li et al., 2023).

#### 4.1.20 PARP

**Fig. 26:**
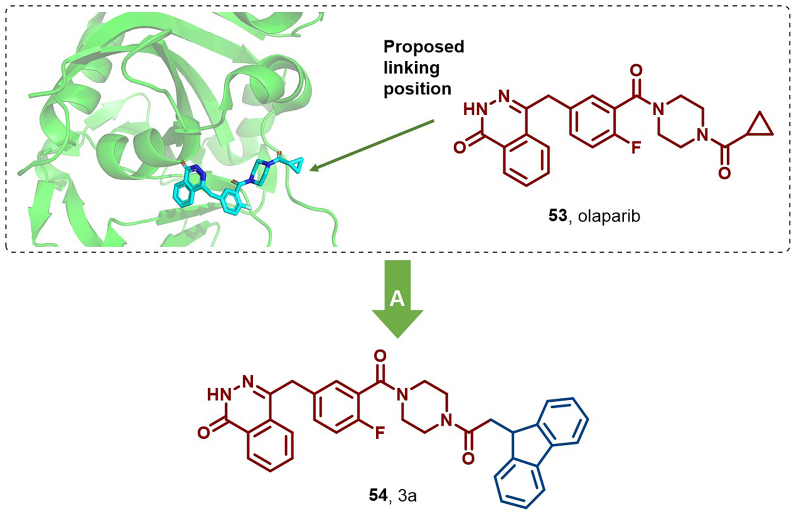
**Molecular structures of PARP1 HyT (54).** A: PARP1 degrader **54** was achieved by PARP1 inhibitor olaparib (**53**) with fluorene, and the crystal structure of **50** in the binding pocket of PARP (PDB: 5DS3).

PARP is a key therapeutic target for cancers harboring mutations in the breast cancer susceptibility genes BRCA 1/2 (Mittica et al., 2018). Olaparib, as a PARP inhibitor, has been clinically employed in the treatment of breast cancer susceptibility gene (BRCA)1/2 mutant cancers (Narod & Foulkes, 2004). However, TNBC presents with poor prognosis and chemotherapy resistance, necessitating new therapeutic strategies. In 2020, Nam et al. successfully synthesized PARP HyTs by conjugating various HyTs to olaparib (**53**) (Go et al., 2020). Notably, HyT 3a (**54**) effectively induced PARP1 degradation in BRCA-mutant breast cancer and TNBC cells, achieving approximately 60% PARP1 degradation in MDA-MB-231 cells at 2.5 μM over 48 h, exhibiting superior antitumor efficacy compared to olaparib (Fig. 26).

#### 4.1.21 Glutathione *S*-transferases (GSTs)

GSTs, crucial phase II detoxification enzymes, are mainly localized in the cytoplasm. They catalyze the conjugation of glutathione with electrophilic exogenous and endogenous compounds, forming water-soluble conjugates readily excreted by cells (Hayes et al., 2005). Thus, GSTs play a fundamental role in cellular defense against environmental stressors, oxidative damage, and drug resistance in cancer cells. Mammalian cytoplasmic GSTs are classified into seven major classes: alpha, mu, pi, theta, sigma, zeta, and omega (Sheehan et al., 2001). Among them, GST-Pi (GSTP) is noteworthy due to its elevated expression in multiple tumors and cancer cell lines, potentially linked to cancer progression and drug resistance (Tew & Townsend, 2011). Given its significance, GSTP is expected to serve as an effective biomarker for early cancer diagnosis, prevention, and treatment (Chatterjee & Gupta, 2018).

**Fig. 27:**
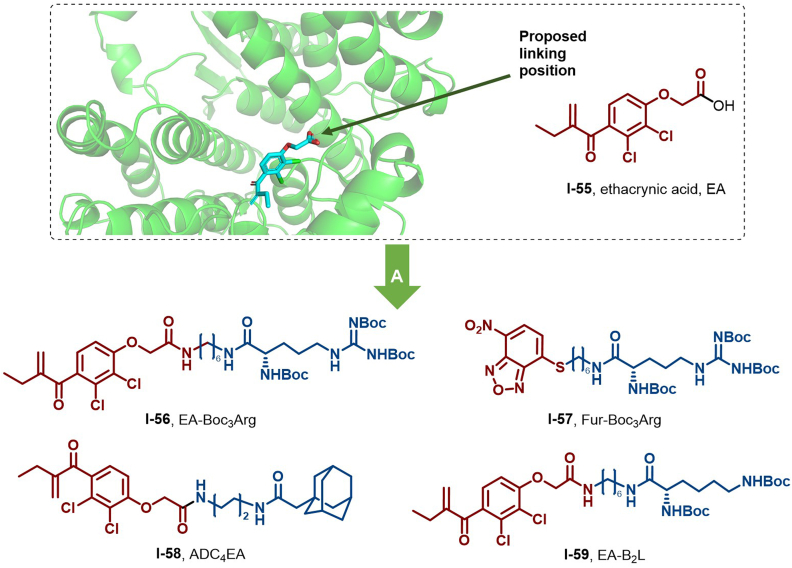
**Molecular structures of GST-HyTs (56–59).** A: GST degraders (**56**–**59**) were obtained by linking the GST covalent inhibitor ethacrynic acid (**55**) with B_3_A, and the crystal structure of **55** in the binding pocket of GST (PDB: 14GS).

To address the overexpression of GSTs in tumor cells, researchers have developed a range of GST inhibitors to augment chemotherapy efficacy (Li et al., 2021). Hedstrom et al. synthesized two HyT molecules, EA-Boc_3_Arg (**56**) and Fur-Boc_3_Arg (**57**), by incorporating Boc_3_Arg into a GST covalent inhibitor (ethacrynic acid, **55**) (Fig. 27). These molecules efficiently degraded GST-α1 and endogenous GSTP in HeLa and NIH_3_T_3_ cells (Long et al., 2012). Furthermore, **56** showed rapid GST-α1 degradation in NIH_3_T_3_ cells (Shi et al., 2016). Mechanistic studies revealed that this degradation is independent of the target protein ubiquitination but relies on the activation of the 20S proteasome *via* the (Boc_3_)-protected arginine (B_3_A) ligand, facilitating B_3_A-dependent target protein degradation.

Subsequently, Niu et al. designed a series of HyT molecules, including ADC_4_EA (**58**), aimed at enhancing GSTP surface hydrophobicity to trigger the cellular PQC mechanism (Fig. 27) (Li et al., 2021). These molecules achieved over 80% GSTP protein clearance at 40 μM and induced GSTP degradation through both the autophagy and ubiquitin-proteasome pathways, presenting a novel therapeutic strategy for cancer. In 2024, Sun et al. successfully identified EA-B_2_L (**59**), an efficient GST degrader, from Boc_2_Lys-conjugated oxalic acid (EA) derivatives. Preliminary degradation mechanism studies guided optimization of the HyT and linker, markedly enhancing GST degradation activity (Sun, Wang, Li, Lü, et al., 2024).

#### 4.1.22 Glutathione peroxidase 4 (GPX4)

**Fig. 28:**
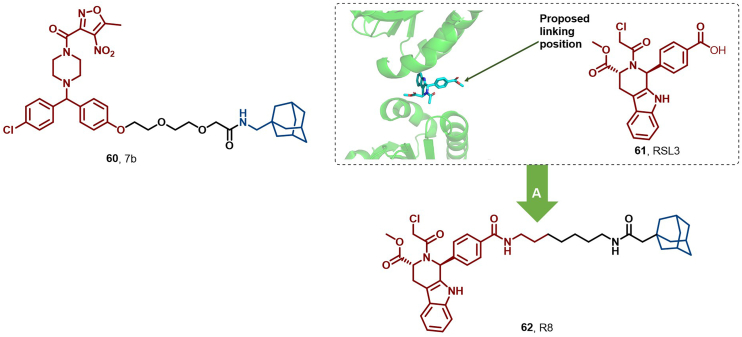
**Molecular structures of GPX4 HyTs (60, 62).** (A) GPX4 degrader **62** was achieved by linking the GPX4 inhibitor RSL3 (**61**) with adamantyl, and the crystal structure of **61** in the binding pocket of GPX4 (PDB: 7U4N).

Ferroptosis, a novel cell death modality, is characterized by iron-dependent lipid peroxidation and is implicated in various diseases, including neurodegenerative disorders and cancer. GPX4 inhibitors induce ferroptosis and hold promise as an anticancer strategy, yet no GPX4 inhibitors have reached the clinical stage due to drug resistance (Li et al., 2024). In 2023, Li et al. reported the first HyT degrader of GPX4, compound **7b** (**60**), which showed potent GPX4 degradation activity (IC_50_ = 0.13 μM, DC_50_ = 0.058 μM) (Fig. 28). Mechanistic studies revealed that **60** degraded GPX4 *via* UPS and the autophagic lysosome system, efficiently induced ferroptosis in HT1080 cells by preventing lipid peroxide removal, thereby showcasing the potential for treating drug-resistant cancers.

In 2024, Ning et al. designed and synthesized a series of HyT degraders by linking the GPX4 inhibitor RSL3 (**61**) to an adamantane moiety. Among them, compound R8 (**62**) was a potent degrader (DC_50_ = 0.019 μM) that efficiently degraded GPX4. **62** exhibited 4-fold enhanced *in vitro* antitumor activity against HT1080 and MDA-MB-231 cell lines compared to the parental compound **61**, with IC_50_ values of 24 nM and 32 nM against HT1080 and MDA-MB-231 cells, respectively. **62** depleted the GPX4 protein mainly *via* the ubiquitin-proteasomal pathway and induced lipid peroxidation (LPO) accumulation. Depletion of GPX4 protein led to LPO accumulation, thereby triggering ferroptosis (Fig. 28) (Ning et al., 2024).

#### 4.1.23 Nucleic acid-binding proteins (NBPs)

NBPs, including DNA-binding proteins (DBPs) and RNA-binding proteins (RBPs), as key regulators of gene expression, show considerable promise as therapeutic targets (Henley & Koehler, 2021; Wu, 2020). However, these proteins were historically considered undruggable due to the absence of small molecule ligand-binding sites (Bushweller, 2019). Despite the potential of HyTs in facilitating protein degradation, their application in DBP and RBP degradation has been hindered by the typical absence of high-affinity small molecule ligands for these proteins (Long et al., 2012; Neklesa & Crews, 2012; Xie et al., 2014).

To address this limitation, Fang et al. developed a novel strategy, termed ligand-assisted covalent hydrophobic tagging (LACHT), designed to target the degradation of DBPs and RBPs. This strategy utilizes *N*-acyl-*N*-alkylsulfonamides (NASA) as an efficient cleavable linker to conjugate a non-covalent POI (protein of interest) ligand with a HyT, thereby enabling protein hydrolytic degradation independently of high-affinity ligands. Through non-covalent protein-ligand orientation, LACHT enables covalent attachment of NASA groups to target proteins *via* hydrophobic adamantane moieties, thereby efficiently labeling them, activating intracellular PQC mechanisms, and ultimately leading to the degradation of the target protein.

**Fig. 29:**
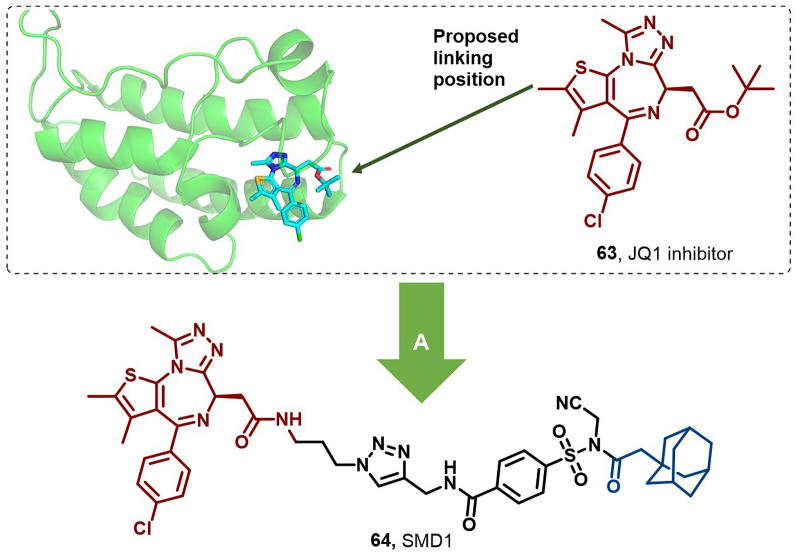
**Molecular structures of ligand-assisted covalent hydrophobic tagging (LACHT) HyTs (64–65).** A: LACHT degrader **65** was obtained by linking the BRD4 inhibitor (**60**) with adamantyl, and the crystal structure of **64** in the binding pocket of BRD4 (PDB: 3MXF).

To validate LACHT's efficacy, Fang et al. selected bromodomain and extra-terminal domain (BET) proteins, which function as DNA-binding transcriptional regulators and play a key role in cancer development, as their study targets (Jin et al., 2022). By incorporating adamantane as a HyT and coupling it with the JQ1 inhibitor (**64**), a non-covalent small-molecule ligand targeting BRD4, they designed a series of small-molecule degraders (SMDs 1–4). Notably, SMD1 (**65**) exhibited the highest efficiency in BRD4 degradation, with a DC_50_ of 3 μM. Further mechanistic studies revealed that BRD4 was first covalently labeled by **65** *via* its hydrophobic adamantane moiety, subsequently underwent polyubiquitination, and was ultimately degraded by the proteasome and lysosome (Fig. 29).

**Fig. 30:**
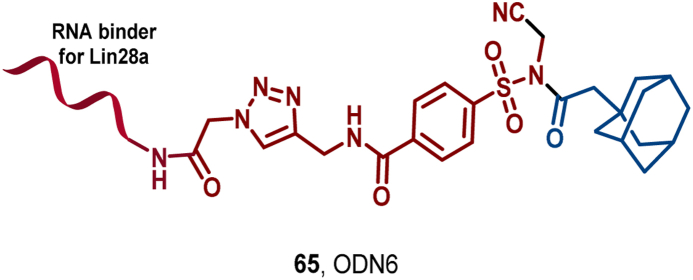
**Molecular structure of LACHT HyT (65)**.

Notably, Fang et al. also extended the LACHT strategy to the degradation of RBPs and found that OND6 (**65**), a degrader targeting the RBP Lin28a, successfully promoted Lin28a degradation by leveraging RNA ligands, resulting in upregulation of its downstream let-7 miRNA (Fig. 30) (Sobral et al., 2016). This finding provides new insights into the application of HyT in RNA-binding protein targeting and further extends the prospects of the LACHT strategy in biomedical applications.

In summary, the LACHT strategy provides a powerful platform for extending the TPD toolbox, especially in biomedical applications.

### 4.2 HyTs for the treatment of neurodegenerative diseases

#### 4.2.1 Alzheimer's Disease (AD)

Neurodegenerative diseases (NDs) constitute a group of heterogeneous disorders characterized by progressive neuronal degeneration or death. The World Health Organization predicts that they will become the second leading cause of mortality within the next two decades (Durães et al., 2018; Fang et al., 2022). NDs include AD, amyotrophic lateral sclerosis (ALS), and Huntington's disease (DiFiglia et al., 1997; Peng et al., 2020; Spillantini et al., 1998; Tiwari et al., 2019).

Despite the absence of a definitive cure for NDs, researchers are vigorously investigating various therapeutic strategies to alleviate symptoms, with strategies including autophagy modulation, miRNA-based therapy, and stem cell therapy (Balestrino & Schapira, 2020; Breijyeh & Karaman, 2020; Van Bulck et al., 2019). These approaches, however, confront challenges such as limited blood-brain barrier permeability, off-target effects, and an inability to target intracellular proteins (Araldi et al., 2020; Rana et al., 2021; Ridolfi & Abdel-Haq, 2017). The continuous advancement of TPD technologies has enhanced cellular permeability, tissue distribution, and pharmacokinetic properties, offering promising prospects for the treatment of NDs.

**Fig. 31:**
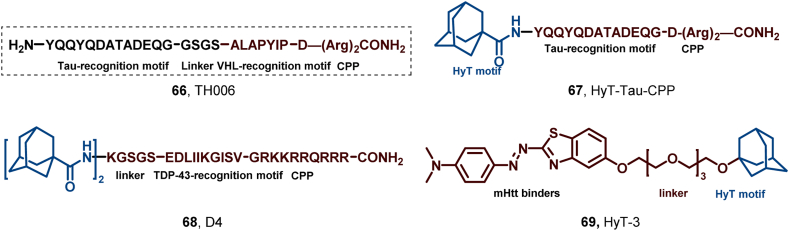
**Molecular structures of tau-targeting peptide-based PROTAC (66), Tau HyTs (67–68), and mHTT HyTs (69)**.

AD, the most prevalent neurodegenerative disease, is marked by memory loss and cognitive decline. Pathologically, it is characterized by β-amyloid plaques and neurofibrillary tangles formed by abnormal tau protein aggregation in the brain. Reducing tau levels may represent an effective strategy for AD. In 2016, Chen et al. pioneered the application of PROTACs in ND treatment, reporting a tau-targeted PROTAC with therapeutic potential in AD (Chu et al., 2016). They designed TH006 (**66**), a tau-targeting peptide-based PROTAC comprising a tau recognition moiety and an E3 ligase-binding moiety to enhance tau degradation (Fig. 31). Tau reduction induced by **66** mitigated Aβ-induced cytotoxicity. However, peptide-based PROTACs exhibited low drug-likeness and limited efficacy.

To develop a more potent degrader, Gao et al. synthesized HyT-tau CPP (**67**) in 2017, a peptidomimetic selective tau degrader (Gao et al., 2017). **67** consists of a HyT, a tau-binding motif, and a cell-penetrating peptide, and it dose- and time-dependently downregulates tau proteins *in vivo* in a proteasome-dependent manner (Fig. 31). Notably, intravenous administration of **67** effectively reduced tau levels in the cerebral cortex of AD model mice, demonstrating its ability to cross the blood-brain barrier.

#### 4.2.2 ALS

ALS, a motor neuron disease, predominantly impacts motor neurons, leading to muscle atrophy and substantial strength reduction. A notable pathological hallmark of ALS is the abnormal accumulation of TDP-43 protein. Consequently, reducing TDP-43 levels has emerged as a promising therapeutic approach (Wils et al., 2010). In 2019, Li and colleagues developed HyT D4 (**68**), a molecule targeting TDP-43, which effectively decreases TDP-43 levels *in vivo* and in a Drosophila model overexpressing TDP-43 (Fig. 31) (Gao et al., 2019). However, significant degradation activity of **68** necessitates high doses (20–150 mM intracellularly), accompanied by mild cytotoxicity.

#### 4.2.3 Huntington's disease

Huntington's disease, an autosomal dominantly inherited neurodegenerative disorder, involves mutations in the HTT gene, resulting in abnormal accumulation of Huntingtin protein, ultimately causing neuronal dysfunction and death (Bates et al., 2015). By 2022, Ishikawa et al. designed and synthesized HyT-3 (**69**) by replacing the E3 ligand portion of mutant HTT-(mHTT-)-targeted **68** with an adamantane moiety. This modification reduced molecular weights and hydrogen bond donors/acceptors (HBD/HBA) and exhibited superior predicted permeability compared to the SNIPER analog (Fig. 31). (Hirai et al., 2022) Furthermore, all HyTs in this study had better membrane permeability-inducing properties compared to the parental SNIPER.

Currently, PROTACs encounter several hurdles in NDs treatment, notably low permeability due to their typically high molecular weights exceeding 800 Da. Transitioning PROTACs to HyTs can mitigate these issues by decreasing molecular mass and HBD/HBA count, thereby enhancing permeability in the central nervous system (CNS) (Fang et al., 2022). Despite HyTs demonstrating substantial therapeutic promise due to their small molecular weight and independence from ubiquitination and E3 ligase, their possible cytotoxicity at high doses necessitates further investigation.

### 4.3 Other disease-related proteins

#### 4.3.1 HaloTag-based systems

**Fig. 32:**
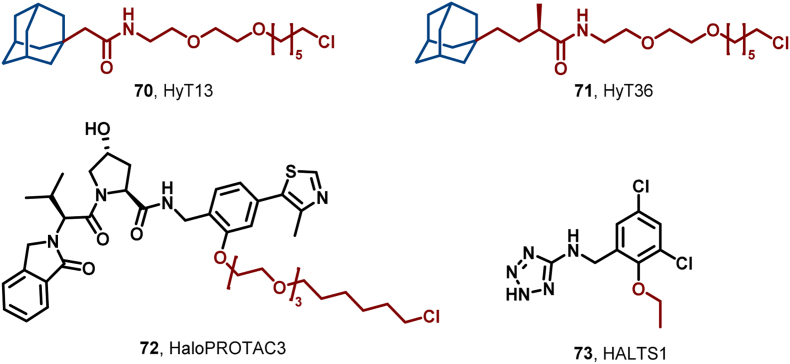
**Molecular structures of Halo-Tag-Based HyTs 70–73**.

HaloTag, a genetically engineered bacterial dehalogenase capable of forming covalent bonds with chloroalkanes, has emerged as a pivotal tool in protein research (Burslem & Crews, 2017). In 2011, Crews et al. reported the potential use of adamantane as a HyT for the degradation of HaloTag fusion proteins in organisms. They synthesized HyT13 (**70**), partially bound to adamantane through a specific linker, which effectively degraded Halo-tag fusion proteins (including HA-EGFP-HaloTag2 and luciferase-HaloTag2, among others) in various systems, such as HEK 293T cells and zebrafish embryos (Fig. 32) (Neklesa et al., 2011).

To enhance HaloTag7 protein degradation, Crews et al. further developed HyT36 (**71**) and HaloPROTAC-3 (**72**) (Fig. 28). Notably, **72** features a bifunctional structure that binds to the VHL E3 ligase ligand on one end and interacts with HaloTag7 fusion protein on the other, facilitating ubiquitination and degradation *via* UPS. In contrast, **71** binds directly to HaloTag7 and undergoes ubiquitination with Hsp70 assistance (Buckley et al., 2015; Tae et al., 2012). Results indicated that **71** degraded HaloTag2 and HaloTag7 more efficiently than **70**, achieving 88% and 65% degradation at 10 μM, respectively. **72** demonstrated even better performance, achieving 90% degradation of GFP-HaloTag7 protein at a concentration of 625 nM, with a DC_50_ value of 19 nM. These findings highlight the potential for further improving HyT degradation activity, crucial for disease control and drug development.

Additionally, HaloTag-2, an earlier version, may cause protein instability under specific conditions, leading to reduced expression of GFP fusion proteins. Neklesa et al. identified HALTS1 (**73**), which binds to the HaloTag-2 core, enhancing thermal stability, reducing ubiquitin-mediated degradation, and increasing fusion protein expression (Fig. 32) (Bushweller, 2019). This HyT/HALTS combination offers a versatile bidirectional system for regulating fusion protein expression through upregulation (HALTS) or downregulation (HyT) within the same cellular context.

#### 4.3.2 Viral proteins

Discovered in the 1950s, RNA-dependent RNA polymerases (RdRps) catalyze the synthesis of RNA templates essential for genome replication and transcription in most RNA viruses. An RdRp consists of three key subunits: PB1, PB2, and polymerase acidic protein (PA)—all involved in “cap-snatching”, where PA cleaves cellular RNA to generate primers for viral mRNA synthesis (Obayashi et al., 2008).

**Fig. 33:**
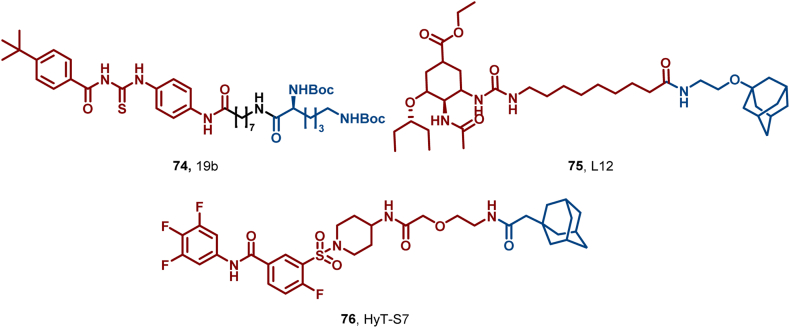
**Molecular structure of PA HyT (74–75), and HBV HyT (76)**.

In 2024, Zhou et al. reported the first acylthiourea-based small-molecule degrader 19b (**74**), targeting influenza virus A/WSN/33/H1N1 PA (Fig. 33). Outperforming corresponding PROTACs, **74** induced robust PA degradation (DC_50_ = 0.06 μM) *via* proteasomal and autophagic pathways. It exhibited extremely high inhibitory activity against A/WSN/33/H1N1 (EC_50_ = 0.015 ± 0.001 μM), suppressed influenza virus replication *in vitro* at low concentrations, and showed efficacy against H3N2, influenza B, and a broad spectrum of enteroviruses (Ma, Wang, et al., 2024). Additionally, Zhou et al. leveraged HyT technology to develop compound L12 (**75**), which markedly enhanced the efficacy of oseltamivir against drug-resistant strains: against the oseltamivir-resistant H1N1-H274Y isolate, **75** achieved an EC_50_ of 0.68 μM—representing a 157-fold improvement over oseltamivir (EC_50_ = 106.8 μM)—with negligible cytotoxicity at 50 μM. **75** selectively degraded viral neuraminidase (NA) while suppressing nucleoprotein (NP) expression (Liu et al., 2025).

Hepatitis B virus (HBV), a hepatotropic DNA virus, causes chronic infection in over 290 million people globally, and existing nucleos(t)ide analogs and core protein allosteric modulators (CpAMs) face challenges of drug resistance and incomplete viral clearance due to HBc mutations. Zhan et al. elucidated the NVR 3–778/HBc complex structure to optimize linkers, yielding HyT-S7 (**76**). **76** exhibited excellent cell activity, degraded HBc *via* the autophagy-lysosome pathway (DC_50_ = 3.02 μmol/L), and effectively degraded 11 HBc mutants—validating HyT for *anti*-HBV therapy (Zhan et al., 2025). These findings pave the way for HyT technology in viral protein degradation, offering a versatile strategy for novel antiviral drug development.

## 5 Conclusions and perspectives

The exploitation of degradation pathways of specific proteins has emerged as a novel therapeutic strategy, particularly for proteins resistant to conventional small molecule inhibitors (Burslem & Crews, 2020). While small-molecule inhibitors demonstrate therapeutic utility in specific contexts, their efficacy is frequently compromised by compensatory protein upregulation and adaptive cellular responses. Current protein degradation technologies (Tsherniak et al., 2017), including PROTACs and MGs, present inherent limitations: PROTAC development is constrained by substantial optimization challenges to achieve drug-like properties, while MG design requires a precise understanding of protein-ligand interaction dynamics (Churcher, 2018; Faust et al., 2020). HyT technology represents a transformative approach that synergizes the modular design advantages of PROTACs with the favorable pharmacokinetic profile of MGs. This innovative strategy offers distinct therapeutic benefits, including enhanced tissue-specific accumulation and the capacity to overcome resistance mechanisms.

Several key challenges currently impede HyT technology development as follows: (1) Mechanistic ambiguity: Incomplete understanding of how HyTs trigger chaperone recognition (e.g., Hsp70/Hsp90 binding specificity) and pathway selection (UPS vs ALP) leads to unpredictable degradation efficiency across different targets. This ambiguity limits the rational design of HyTs for novel proteins, as degradation outcomes cannot be reliably inferred from target structure alone; (2) Optimization of linker and structural diversity bottlenecks: Existing HyT linkers are often limited to short alkyl chains or rigid scaffolds, restricting tuning of HyT exposure and target-protein binding kinetics. Poor linker optimization causes either weak target engagement (reducing degradation potency) or non-specific hydrophobic interactions (increasing off-target effects), thus hindering *in vivo* efficacy; and (3) Identification of novel targets and ideal HyTs: Most HyTs focus on well-characterized oncoproteins (e.g., ALK, CDK9), while challenging targets (e.g., membrane proteins, multi-protein complexes) remain underexplored due to the difficulty in designing tags that access hydrophobic regions. Additionally, current HyTs lack structural diversity and exhibit poor oral bioavailability (Churcher, 2018).

To address the aforementioned challenges, targeted strategies rooted in structural biology, chemical engineering, and translational technology can be implemented, as detailed below: (1) Systematically explore novel E3 ubiquitin ligase types and their mediated degradation mechanisms compatible with HyT technology using activity-based protein profiling (ABPP)-based fragment screening, multi-omics integrative analysis, clustered regularly interspaced short palindromic repeats/CRISPR-associated protein 9 **(**CRISPR/Cas9) gene editing technology, or pull-down assays. (2) Modular chemistry and high-throughput screening: Adopt “click chemistry” for rapid synthesis of linker libraries with varied lengths, flexibility (e.g., alkyl vs polyethylene glycol scaffolds), and polarity; Develop macrocyclic or hydrophilic-hydrophobic hybrid HyTs (e.g., adamantane-polyethylene glycol conjugates) to expand structural diversity; Integrate computational docking to preselect tags with optimal fit to target hydrophobic pockets, reducing non-specific interactions. (3) Expanding target scope and improving druggability. Leverage multi-omics data (e.g., phosphoproteomics of drug-resistant tumors) to prioritize underexplored targets (e.g., membrane-associated kinases, disordered/aggregate-prone targets, multipass membrane proteins, organelle-localized proteins, and scaffolding proteins in multi-protein complexes). For membrane targets, design membrane-penetrating HyTs to access intracellular hydrophobic regions. Optimize formulations using lipid-based drug delivery systems to improve HyT solubility and bypass hepatic first-pass metabolism. Alternatively, conjugate HyTs with cell-penetrating peptides to enhance oral absorption while maintaining target specificity.

As an emerging therapeutic modality, HyT-PD represents a novel source of targeted therapeutic molecular degraders (Békés et al., 2022). However, the field faces substantial knowledge gaps regarding fundamental degradation mechanisms, necessitating further in-depth research into the following key directions: (1) Novel tag and scaffold innovation: Develop AI-driven predictive computational models for structure-based rational HyT design; engineer “smart HyTs” with stimuli-responsive properties to achieve spatiotemporal degradation control; (2) Mechanistic exploration: Elucidate the underlying mechanism of novel HyTs *via* a systematic screening framework (ABPP fragment screening, CRISPR/Cas9-mediated gene editing, and pull-down assays) to expand degradation pathways and enable rational HyT engineering; and (3) Translational breakthroughs: Advance preclinical candidates to investigational new drug (IND)-enabling studies, with a focus on CNS disorders and drug-resistant tumors where HyT's permeability advantage is prominent. Unlocking the full potential of HyT-PD will require multidisciplinary collaboration across chemical biology, structural biology, and clinical oncology to advance our understanding of degradation mechanisms, discover next-generation HyT molecules, and translate these findings into clinically effective therapeutics.

## CRediT authorship contribution statement

**Lilan Xin:** Writing – review & editing, Writing – original draft. **Hongli Wang:** Writing – original draft. **Maoze Yang:** Writing – original draft. **Zhangxiao Guo:** Writing – original draft. **Man Zhu:** Writing – original draft. **Terry W. Moore:** Writing – review & editing, Conceptualization. **Chune Dong:** Writing – review & editing, Conceptualization. **Hai-Bing Zhou:** Writing – review & editing, Conceptualization.

## Declaration of competing interest

The authors declare that they have no known competing financial interests or personal relationships that could have appeared to influence the work reported in this paper.
